# Supplementary material for: Complexome profiling of the Chlamydomonas psb28 mutant reveals TEF5 as an early PSII assembly factor
Source: Plant Cell. 2025 Mar 17;37(6):koaf055. doi: 10.1093/plcell/koaf055 (PMC12164590; doi:10.1093/plcell/koaf055)
Supplement: koaf055_Supplementary_Data [file koaf055_supplementary_data.zip › psb28_interactive.zip!/plots/intensity/qualityPlots/peptides/CPLD38_Cre01.g000850.t1.2.html]

### Cre01.g000850.t1.2

Trivial name: CPLD38  
SeqCoverage [median]: 31.10  
Mass: 20.69 kDa  
  
 

| Sequence | MissedCleavage | Mass [Da] | wtIntensity | muIntensity | Score |
| --- | --- | --- | --- | --- | --- |
| ASNEEKEVQYNK | 1 | 1437.67 | 3614.45 | 2481.26 | 98.11 |
| EFGYSR | 0 | 757.34 | 0.00 | 759.09 | 79.45 |
| EVQYNK | 0 | 779.38 | 749.09 | 2700.55 | 101.72 |
| KRVEEMTEAELEQLASEVDADKQR | 3 | 2803.37 | 0.00 | 4486.83 | 95.93 |
| QLEQYEEAVMR | 0 | 1394.65 | 7400.10 | 8324.79 | 212.40 |
| QLEQYEEAVMRK | 1 | 1522.74 | 6645.53 | 5772.33 | 122.55 |
| QMTYVK | 0 | 768.38 | 1484.58 | 3352.01 | 144.29 |
| RVEEMTEAELEQLASEVDADKQR | 2 | 2675.27 | 0.00 | 7141.01 | 97.78 |
| VEEMTEAELEQLASEVDADKQR | 1 | 2519.17 | 0.00 | 2865.82 | 130.87 |
|  |  |  | 19893.8 | 37883.7 |  |
